# Many LINE1 elements contribute to the transcriptome of human somatic cells

**DOI:** 10.1186/gb-2009-10-9-r100

**Published:** 2009-09-22

**Authors:** Sanjida H Rangwala, Lili Zhang, Haig H Kazazian

**Affiliations:** 1 Department of Genetics, University of Pennsylvania School of Medicine, Hamilton Walk, Philadelphia, Pennsylvania 19104, USA

## Abstract

**Background:**

While LINE1 (L1) retroelements comprise nearly 20% of the human genome, the majority are thought to have been rendered transcriptionally inactive, due to either mutation or epigenetic suppression. How many L1 elements 'escape' these forms of repression and contribute to the transcriptome of human somatic cells? We have cloned out expressed sequence tags corresponding to the 5' and 3' flanks of L1 elements in order to characterize the population of elements that are being actively transcribed. We also examined expression of a select number of elements in different individuals.

**Results:**

We isolated expressed sequence tags from human lymphoblastoid cell lines corresponding to 692 distinct L1 element sites, including 410 full-length elements. Four of the expression tagged sites corresponding to full-length elements from the human specific L1Hs subfamily were examined in European-American individuals and found to be differentially expressed in different family members.

**Conclusions:**

A large number of different L1 element sites are expressed in human somatic tissues, and this expression varies among different individuals. Paradoxically, few elements were tagged at high frequency, indicating that the majority of expressed L1s are transcribed at low levels. Based on our preliminary expression studies of a limited number of elements in a single family, we predict a significant degree of inter-individual transcript-level polymorphism in this class of sequence.

## Abstract 2

Over 600 LINE 1 elements are shown to be transcribed in humans; 400 of these are full-length elements in the reference genome.

## Background

The human genome is littered with retrotransposons: roughly 20% of genome sequence is derived from LINE1 (L1) elements. Autonomous L1s are approximately 6,000 bp in size and encode two open reading frames (ORFs): ORF1, an RNA-binding protein that functions as a nucleic acid chaperone [1], and ORF2, a reverse transcriptase [2] and endonuclease [3]. Both of these proteins are critical for retrotransposition [4]. There are approximately 7,000 full-length elements in the human reference genome, 304 of which belong to the most recently evolved L1Hs subfamily [5,6].

Full-length human L1 elements contain a conserved 5' untranslated region (UTR) of approximately 900 bp that carries an internal RNA polymerase II promoter [7]. Binding sites for *RUNX3 *[8], *SRY *[9] and *YYI *[10,11] within the first few hundred base pairs of this UTR are important for optimal expression of the transcript. In addition, *YY1 *activity promotes transcriptional initiation from the start of the element [10], although Lavie *et al*. [12] found that transcripts could also initiate upstream or downstream depending on the context of upstream non-L1 sequence. L1s propagate through reverse transcription of this primary transcript and integration into the genome [13,14]. This process is inefficient, so that the majority of product is 5' truncated, containing only a 3' portion of the element [15]. The human genome contains on the order of 500,000 non-autonomous, truncated elements [6].

While older and truncated elements have lost the ability to retrotranspose, at least some of the more evolutionarily recent elements are active, as evidenced by the high number (approximately 500) of polymorphic insertion sites found in human populations (compiled in [16]), many of which have contributed to the etiology of human diseases (reviewed in [17,18]). At least 40 of the human-specific subfamily L1 elements in the haploid reference genome were found to be competent for retrotransposition in a cell culture assay [19]. L1s that can no longer mobilize themselves may also be significant. L1s are also responsible for the *trans*-mobilization of non-autonomous sequences such as Alus, SVAs, and even cellular RNAs to produce processed pseudogenes [20]. *Trans*-mobilization may not require active ORF1 [21] and so might be carried out by a partially degenerate, yet transcribed, L1. Elements that have lost function for both ORF1 and ORF2 may still contribute promoter and polyadenylation sites that can interfere with the transcriptional regulation of a genomic region [22,23]. For instance, transcription through an older element on human chromosome 10 appears to be involved in the formation of a neocentromere [24]. L1s also might be important in recruiting DNA methylation and heterochromatin formation on the inactive X chromosome [25]. In plants, the presence of transcription through a retrotransposon results in altered regulation of neighboring genes [26].

L1s in somatic tissues have been thought to be mainly quiescent: neither transcribed nor retrotransposing, rendered silent by cytosine methylation [27-30] and histone modification [31]. Those L1s that are expressed are often prematurely aborted through internal splicing or polyadenylation [32,33]. Yet, growing evidence questions the assumption that all L1s are suppressed: L1s may in fact be both transcribed and mobile, not just in the germline [34-36], but also in the early embryo [37], and in certain other tissues [38-40]. It is unclear how many of the thousands of L1 promoters in the genome are active, as sequences derived from repetitive DNA are typically excluded from most genome-wide transcriptome analyses (see [41] for a recent exception).

We were interested in the number and nature of L1 elements that contribute to the transcriptome of human somatic cells. Because the human genome contains over 100,000 sequences that are nearly identical in sequence, it is often impossible to identify the particular insertion site from amplicons located within the element. Flanking sequence, in some cases only a few bases, is necessary in order to determine the genomic location of an element. We have used variations on 3' and 5' rapid amplification of cDNA ends (RACE) in order to trap flanking sequence tags specifically from expressed human L1 elements. Below, we describe our results, which have revealed 692 distinct loci, 410 of which correspond to full-length retroelements in the human reference genome.

## Results

### Isolation and characterization of L1 expression tags from lymphoblastoid cell lines of humans

#### Isolation of 3' expression tags derived from particular transcribed L1 loci

While L1s carry adequate information for transcriptional termination and polyadenylation [42], the polyadenylation site is non-canonical, so that L1 transcripts often do not end exactly at the end of the element [43]. This is manifest in the number of L1 elements carrying 3' transduced sequence from their progenitor locus: about 10% of all retrotransposition events [44-47]. We predicted that a small proportion of all transcripts from expressed L1s would carry non-L1 sequence resulting from read-through of the transcript into the flanking genomic region. These sequences could then be used to identify the genomic location of the element. In some cases, the terminal few bases of the L1 3' UTR might be sufficient in themselves to locate the element uniquely in the human reference genome.

**Figure 1:**
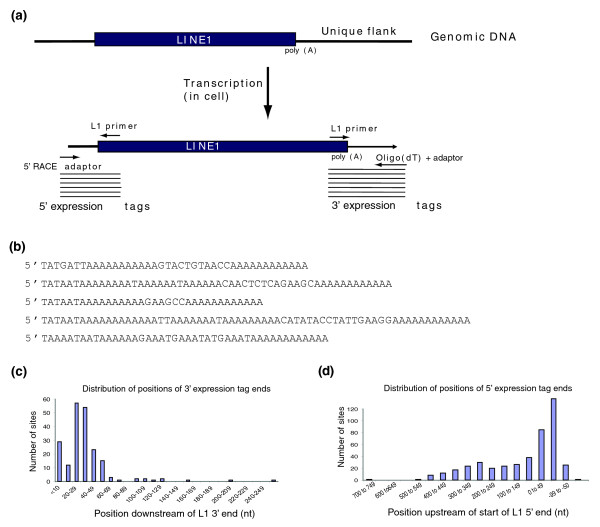
Description of data collection method and overview of results. **(a) **Diagram of expression tag capture. L1 elements are often naturally transcribed with non-L1 sequences at the 5' and/or 3' end. A 3' RACE adaptor/oligo(dT) primer and L1 specific primer can be used to capture expressed sequence from the 3' end. Similarly, a 5' RACE adaptor and L1 specific primer can capture 5' start sites that occur in non-L1 sequence. PCR is subsequently used to amplify the signal from the expressed tags. **(b) **Examples of 3' expression tags. Sequences start at the end of the L1 and terminate in 12 adenosines derived from the 3' RACE adaptor. **(c) **Histogram depicting the distribution of polyadenylation positions of 3' expression tags, relative to the end of the L1. **(d) **Histogram depicting distribution of 5' start site positions upstream of the 5' end of full-length L1 elements. Negative start sites occur in the 5' UTR downstream of the consensus 5' end of the element. Nt, nucleotides.

We primed first strand synthesis of cDNA using oligo(dT), followed by second strand synthesis with an oligonucleotide located at the end of the 3' UTR of the L1 (Figure 1a). Due to the LINE1-associated poly(A) tract, 3' end sequence amplicons tend to be of low complexity (Figure 1b). We have been unsuccessful in obtaining adequate sequence quality and length from these amplicons using next generation sequencing methodologies; below, we describe our results using manually curated sequence reads that were generated by the Sanger method.

We obtained 3' end transcript sequence from 2,152 cDNA clones from lymphoblastoid cell lines from a single European-American individual, GM10861, from the Centre d'Etude du Polymorphisme Humain (CEPH) population (Table 1). Nearly half of these expressed sequence tags had been primed from the polyadenylation site immediately downstream of the L1, and therefore aligned to multiple identical L1 3' UTR locations in the genome. However, 1,148 expression tags were unique in the reference genome; these represented 204 distinct sites, 54 of which corresponded to full-length L1 elements. Thirty-eight L1 expression tag clusters could not be mapped adjacent to an L1 in the reference genome.

**Table 1:** Summary of sequencing analysis of 3' and 5' L1 expression tags

**Cell line**	**Amplicons sequenced**	**Expression tags to unique sites**	**Tagged sites**	**Sites not associated with a reference L1**	**Tagged full-length L1 elements**
3' RACE: GM10861	2,152	1,148	204	38	54
3' RACE: total	3,828	1,592	271	43	69
5' RACE: GM11994	36,088	14,488	427	4	347
Total	39,916	16,080	692	47	410

Expression tags were typically short (Figure 1b, c; Additional data file 1), with a mean end position of 34 nucleotides from the end of the L1 (median = 30.5 nucleotides). Seventy-five percent (152) of tagged sites terminated transcription less than 40 nucleotides from the end of the L1, and 93% (190) terminated less than 60 nucleotides from the L1 (Figure 1c). The distribution of polyadenylation positions for expression tags corresponding only to full-length elements was similar (mean = 35 nucleotides; median = 29 nucleotides; 81% located less than 40 nucleotides from the L1). Many of these short transductions represent non-canonical L1 3' ends or atypical polyadenylation cleavage sites, rather than the use of novel polyadenylation signals downstream of the L1 itself.

Thirty-seven L1 elements were tagged five or more times (Additional data file 2); these include six full-length elements, two containing intact, putatively functional ORFs (Table 2, 4p15.32 and 7q31.1). The Chao2 ecological index, which estimates the number of types based on the rate of sampling singletons and doubletons [48], predicts a total of 363 expressed sites in this individual. As over half of the 204 sites we identified are represented by only one or two expression tags, it is likely that increased sequencing will yield few significantly expressed new sites.

**Table 2:** Full-length L1 elements identified by 3' expression tag analysis

**Chromosome band**	**Sub-family**	**Genome coordinates (hg18)**	**ORF1, ORF2**	**Tag count**	**In intron of gene, +/-**	**dbRIP ID **[16]	**Identified by 5' expression tag, count**
1p21.1	L1HS	chr1:105187979-105194009	-, -	1	No		
1q25.2	L1PA2	chr1:177073306-177079473	-, -	3	No		
1q44	L1PA3	chr1:246757382-246763965	-, -	2	No		Yes, 3
1p31.1	L1PA2	chr1:83588685-83594736	-, -	1	No		
1p22.3	L1HS	chr1:86917352-86923382	Yes, Yes	1	No		
2q23.1	L1HS	chr2:148662785-148668812	Yes, -	1	Yes, +		
2p24.3	L1HS	chr2:16638475-16644507	Yes, Yes	1	Yes, +		
2q31.1	L1HS	chr2:169813380-169819412	Yes, -	4	Yes, -		
2q31.3	L1HS	chr2:181406634-181412661	-, Yes	3	No		
2q34	L1HS	chr2:214140201-214146231	Yes, -	5	Yes, -		
2q37.1	L1HS	chr2:232722151-232728183	-, -	1	Yes, -		Yes, 1
2p16.2	L1PA3	chr2:53667675-53673685	Yes, -	1	No*		
2p13.3	L1HS	chr2:71492113-71498139	Yes, -	2	Yes, -		
3q12.2	L1PA3	chr3:101711142-101717175	-, -	1	Yes, +		
3q13.32	L1PA2	chr3:120115781-120121808	-, -	1	Yes, -		
3q13.33	L1PA2	chr3:123243449-123249475	Yes, -	4	No		
3p24.3	L1PA3	chr3:18992446-18998582	-, -	1	No*		
3p24.3	L1PA3	chr3:23365739-23371880	Yes, -	1	Yes, +		Yes, 71
4q27	L1HS	chr4:121089330-121095361	Yes, -	2	No*		
4q31.22	L1HS	chr4:145977329-145983590	-, -	1	No		
4p15.32	L1HS	chr4:15452268-15458293	Yes, Yes^†^	349	Yes, +		
4q13.1	L1PA2	chr4:64080835-64086866	-, -	6	No		Yes, 30
4q23	L1HS	chr4:99732610-99738637	Yes, -	1	Yes, +		
5q23.2	L1PA3	chr5:126253878-126259924	Yes, -	4	Yes, +		
5q34	L1PA6	chr5:162571721-162577711	-, -	3	No*		
5q35.3	L1PA2	chr5:180262128-180268143	Yes, -	2	Yes, -		
5p13.3	L1HS	chr5:34183708-34189893	Yes, -	1	No	Druze54	
5q14.1	L1PA3	chr5:77910921-77916574	-, -	1	Yes, +		
6q22.31	L1PA5	chr6:125758770-125765089	-, -	1	No		
6p22.2	L1HS	chr6:24919886-24925913	Yes, Yes	1	Yes, +	AL512428| Database 29^‡^	
6q13	L1HS	chr6:70776961-70783165	Yes, -	17	Yes, +	L1HS169	
6q14.3	L1HS	chr6:86765484-86771510	Yes, -	2	No	chr6-8676	
6q15	L1PA3	chr6:88089716-88095715	-, -	1	Yes, -+		
7q31.1	L1HS	chr7:110670808-110676838	Yes, Yes	5	Yes, +	^‡^	
7q31.1	L1HS	chr7:113203414-113209443	Yes, Yes	13	No	^‡^	
7q36.1	L1PA5	chr7:149925116-149930649	-, -	17	No		
7p14.3	L1PA2	chr7:32703791-32709682	-, -	2	No*		
7p12.1	L1PA2	chr7:50934034-50940065	Yes, -	1	No*		
8p21.2	L1PA2	chr8:26309046-26315012	Yes, -	12	Yes, +		
8q21.13	L1PA2	chr8:84521933-84527959	Yes, -	4	No		
8q22.1	L1PA2	chr8:96633637-96639669	Yes, -	2	No*		
9q31.3	L1HS	chr9:112593199-112599230	Yes, -	2	Yes, +		
9q21.11	L1PA2	chr9:71281844-71287865	-, -	1	Yes, -		
9q22.32	L1HS	chr9:95915639-95921668	Yes, -	2	No		
10q26.12	L1PA3	chr10:122660462-122666485	-, -	2	No		Yes, 10
10p15.1	L1HS	chr10:6451604-6457635	-, Yes	2	No	L1HS171| Database 45	
11q22.3	L1HS	chr11:108553432-108559463	Yes, Yes	1	No		Yes, 1
11p15.4	L1PA2	chr11:7635956-7641978	-, -	19	No		
12q23.3	L1PA2	chr12:105389774-105395799	Yes^†^, -	1	Yes, -		
12q24.32	L1PA3	chr12:126916354-126922380	-, -	3	No		
12q13.13	L1HS	chr12:50242683-50248708	Yes, -	2	No		
12q21.1	L1PA2	chr12:72074857-72080877	-, -	2	No		
12q23.1	L1PA2	chr12:95233852-95239880	-, -	5	Yes, -		
13q12.3	L1HS	chr13:30774452-30780482	Yes, -	1	Yes, +		
13q13.3	L1PA3	chr13:36722478-36728518	-, -	2	No		
13q14.2	L1HS	chr13:47937693-47943703	-, -	6	Yes, +		
13q21.32	L1PA2	chr13:67152381-67158421	-, -	4	No		
14q23.1	L1PA2	chr14:59482976-59488994	-, -	1	Yes, -		
14q31.1	L1PA3	chr14:79303855-79309939	-, -	1	Yes, +		
16q22.1	L1HS	chr16:67174881-67180909	Yes, -	1	No		
20p11.21	L1HS	chr20:23354746-23360777	Yes, -	2	No*		
20q13.2	L1PA2	chr20:51553798-51559820	-, -	1	No*		
22q11.22	L1PA3	chr22:20,961,183-20,967,196	-, -	1	Yes, -		
22q12.1	L1HS	chr22:27389272-27395303	Yes, Yes	2	No*	L1HS86| AL121825^‡^	
Xq26.1	L1PA2	chrX:129920587-129926612	Yes, -	2	No		
Xq27.2	L1HS	chrX:141393302-141399320	Yes, Yes	2	No	AL031586	
Xp21.3	L1PA2	chrX:28134830-28140827	-, Yes	1	No		
Xp11.22	L1PA2	chrX:49711541-49717572	Yes, -	1	Yes, +		
Xq13.2	L1PA4	chrX:73611039-73617191	-, -	1	Yes, -		

*Spliced ESTs span L1. ^†^Truncated 96 amino acids from carboxyl terminus.

^‡^Active (>1% L1RP) in cell culture assay [19].

We have also obtained 3' sequence tags from five additional individuals: GM17032 and GM17033 are African-Americans, GM17045 is of Middle Eastern origin, and GM11994 and GM11995 are European-American individuals who are the parents of GM10861 described above. In total, 3,828 3' expression tags were sequenced from all six individuals (Table 1; Additional data files 1, 2 and 3), encompassing 1,592 sequences corresponding to 271 unique sites. Of these sites, 228 corresponded to an L1 element in the reference genome. The remaining 43 clusters, while containing L1 3' UTR sequence at one end, do not map to any of the reference L1s, and, therefore, may represent private or polymorphic insertions. Due to the extremely short, homopolymeric nature of these tags, we cannot map the putative location of these 43 clusters in the reference genome or design PCR oligonucleotides to verify their presence in genomic DNA.

Forty-seven L1 sites were sampled five or more times, while 26 were sampled ten or more times. These relatively highly expressed sites include ten full-length elements (Additional data file 1). Expression tags corresponding to different elements were cloned from different lines, and no elements were cloned from all six lines (Additional data file 3). We focused our interest on full-length elements, which might be transcribed from the native promoter in the 5' UTR and could potentially produce active ORF1 and/or ORF2 protein. Sixty-nine full-length elements in the human reference genome were identified in our 3' expression tag analysis (Table 2), which is significantly greater than the proportion of full-length elements in the reference genome from the Pa7 family or younger (Fisher's exact test *P *= 1.0 × 10^-15^). Of the full-length elements tagged, more are from the human specific subfamily (30) than their proportions in the genome (Fisher's exact test *P *= 7.8 × 10^-21^); however, this is not surprising because the primers that were used contained a nucleotide at the 3' end that biased amplification towards the L1Hs human specific subfamily.

Of the 69 expressed full-length elements, 30 are present in genes (Table 2), which is somewhat more than expected from the proportions in the genome (Fisher's exact test *P *= 0.0013). Of the elements present within genes, slightly more than would be expected by their distribution in the genome are in the same orientation as the gene (Fisher's exact test *P *= 0.0026; L1Hs only, Fisher's exact test *P *= 0.017; Table 2). This is in keeping with the possibility that some of these L1s may be expressed as a side effect of transcription of the host gene.

Seven expressed full-length elements contain intact *ORF1 *and *ORF2 *and might be competent for retrotransposition under certain conditions. Four additional elements contain potentially active *ORF2 *in the absence of *ORF1 *(Table 2). The proportions of expression tagged elements from the L1Hs subfamily containing intact *ORF1*, *ORF2*, both or neither are not significantly different from those present in the genome as a whole (χ^2 ^= 2.36, degrees of freedom = 3, *P *= 0.5).

#### Isolation of 5' expression tags that identify transcriptional start sites of transcribed L1 elements

To supplement our 3' end analysis, we also conducted L1 5' RACE on RNA from lymphoblastoid cell lines corresponding to a single European-American individual, GM11994, the father of GM10861 described above. Expression tags obtained using 5' RACE identify L1 transcription start sites, either from the native L1 promoter or from an upstream promoter (Figure 1a). As the 5' end of a full-length L1 is not homopolymeric, we were able to obtain high quality reads using high-throughput 454 pyrosequencing. We recovered 36,088 sequences, of which 14,488 corresponded to 427 locations in the reference genome (Table 1; Additional data file 4). The Chao2 index predicts 494 sites in total; therefore, these loci include the majority of the expressed sites within this particular individual, and likely include all the highly expressed sites.

Only six of the full-length 5' RACE expression-tagged L1 elements were also found by 3' expression tagging (Table 2). This lack of overlap is instructive, though not entirely surprising, as 3' tags would include both full-length and 5' truncated elements, the latter being the most common in the genome. In contrast, 5' RACE is biased towards full-length elements, as relatively few L1s are 3' truncated. Moreover, the oligonucleotide used to prime 3' amplification contained a nucleotide change that biased it towards amplification of the L1Hs subfamily, whereas the 5' RACE primer was unbiased and would identify all L1 5' UTR-derived sequence.

We identified 347 sites corresponding to full-length expressed elements by 5' RACE analysis, 89 of which were sampled 10 or more times (Additional data file 4). Of the remaining expressed sites, 76 corresponded to deleted or degenerated 3' truncated elements from the L1P1, L1P2 and L1P3 subfamilies (Additional data file 4, grey font). Four tagged sites did not correspond to an L1 element in the reference genome (Additional data file 4, blue font). We were able to verify by PCR that one of these four sites, which mapped to chr12: 33908761, identifies a non-reference L1 present in the GM10861/GM11994/GM11995 familial trio. The precise insertion breakpoint of this L1 was determined by sequencing of the PCR verification product (Additional data files 4 and 5).

L1 5' start sites mapped by 5' RACE can be subdivided into three groups: those that are located in the upstream flanking sequence, those that are internal to the element, and those that splice from far upstream. Four expression tags indicated usage of a promoter far upstream (>15 kb) that produced a transcript that spliced immediately adjacent to a full-length L1 (Additional data file 4, green font). Of the start sites mapping internally or within 1,000 bp upstream of a full-length L1, 50% (170) were located within ± 50 nucleotides of the consensus start of the L1 (Figure 1d), with the median start site at position -21 relative to the L1. These relatively close, though variant, start sites are typical of usage of the native L1 promoter [12]. However, 124 5' expression tags to full-length elements begin greater than 100 nucleotides upstream of the L1 (Figure 1d), suggesting that a proportion of L1 transcripts from certain loci might also originate from upstream flanking promoters.

Of the full-length elements identified, 24 are from the L1Hs human-specific subfamily, which is not significantly greater than what would be expected based upon the proportions found in the genome (Fisher's exact test *P *= 0.26; Table 3). However, elements from the next youngest L1Pa2 (Fisher's exact test *P *= 9.9 × 10^-13^) and L1Pa3 (Fisher's exact test *P *= 1.7 × 10^-10^) subfamilies are overrepresented, while the older L1Pa5 (Fisher's exact test *P *= 2.6 × 10^-5^) and L1Pa6 (Fisher's exact test *P *= 5.6 × 10^-15^) elements are underrepresented. This is consistent with the hypothesis that more evolutionarily recent elements are more likely to have retained sequences that would be permissible for transcription. Of the 24 full-length L1Hs elements, eight contain intact *ORF1 *and *ORF2*, two contain an intact *ORF2 *only, and nine contain an intact *ORF1 *only (Table 3). Relative to the proportions in the genome, the distribution of elements containing intact *ORF*s is not significant (χ^2 ^= 0.7, degrees of freedom = 3, *P *= 0.9).

**Table 3:** Full-length L1Hs subfamily elements identified through 5' expression tag analysis

**Chromosome band**	**Genome coordinates (hg18)**	**ORF1, ORF2**	**dbRIP ID **[16]	**Position of 5' tag start relative L1 5; end (nt)**	**Tag count**
1q31.3	chr1:194455124-194461155	Yes, -		-380	3
2q12.1	chr2: 102549247-102555276	Yes, Yes		-1	1
2q24.1	chr2:158131112-158137135	-, -		-77	9
2q37.1	chr2:232722151-232728183	Yes, -		-456	1
3p24.3	chr3:18946979-18953016	-. -		+42	1
3q25.32	chr3:159220160-159226187	Yes, -		0	2
4q21.21	chr4:79245914-79251943	Yes, -		-2	6
5q14.1	chr5:79110459-79116517	Yes, Yes	*	-59	1
5q14.3	chr5:85842264-85848292	Yes, Yes		-364	1
6q14.2	chr6:84100391-84106433	-, Yes		-4	4
7q35	chr7:147170579-147176648	-, -		-322	6
8q24.13	chr8:126664313-126670315	Yes, Yes	238261	-3	5
10q25.1	chr10:107127095-107133125	-, Yes		+50	8
11q14.3	chr11:90339088-90345118	-, -		+44	4
11q21	chr11:92509453-92515487	Yes, Yes		-394	2
11q22.3	chr11:108553432-108559463	Yes, Yes		+50	1
12q24.32	chr12:125349470-125355537	Yes, Yes	*	-340	5
13q12.3	chr13:29113844-29119843	Yes, Yes	L1HS235| Database39	-1	1
14q12	chr14:30223767-30229794	Yes, -		+67	1
14q22.1	chr14:51331070-51337100	Yes, -		-392	1
15q25.2	chr15:81910561-81916590	Yes, -		-7	1
16q21	chr16:64281416-64287424	Yes, -		-119	2
18p11.21	chr18:13965860-13971890	-, Yes		-2	4
20q13.2	chr20:53868030-53874021	Yes, -	237994	-474	2

*Highly active (>1% L1RP) in cell culture assay [19].

### Further characterization of selected expression-tagged L1 elements indicates inter-individual differences in transcript levels

#### The L1 at 4p15.32 is the progenitor of transduced daughter elements

**Figure 2:**
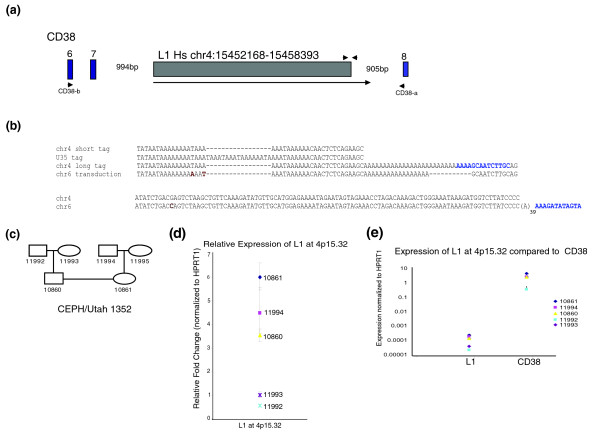
Characterization of L1 at 4p15.32. **(a) **Diagram of L1 at 4p15.32 and the surrounding region. The arrow designates the L1 transcript. Blue boxes indicate exons of the *CD38 *gene, with exon number designated. Oligonucleotides CD38-a and CD38-b are indicated. Unmarked triangles indicate the positions of oligonucleotides used in L1 TaqMan qPCR assay. **(b) **Alignments of L1 at 4p15.32 3' end and related sequences. 'chr 4 short tag' - the major 3' expression tag cloned from this site. 'chr4 long tag' - longer 3' expression tag and 3' RACE sequence cloned from this site. 'chr6 transduction' - paralogous, transduced sequence downstream of L1 on chromosome 6. 'U35' - similar distinct 3' expression tag that cannot be mapped to the human reference genome. 3' end target site duplications are highlighted in blue. Single nucleotide differences in the chromosome 6 sequence are highlighted in dark red. **(c) **Diagram of the pedigree of the CEPH/UTAH individuals used in this study. **(d) **Relative expression of the L1 at 4p15.32 in lymphoblastoid cell lines from CEPH individuals. Expression is in arbitrary units normalized to HPRT1. Error bars indicate ± standard deviation from three replicates. **(e) **Expression of CEPH individuals of the L1 at 4p15.32 compared to flanking exons of *CD38*, normalized to HPRT1. Expression is plotted on a logarithmic scale so that levels for both amplicons can be clearly visualized. Error bars represent ± standard deviations from three replicates. All data are representative of at least two biological replicates.

We have characterized the nature of transcription from four full-length elements identified by 3' expression tags. The most frequent 3' expression tag (Table 2; Additional data file 1) we identified corresponds to an element from the L1Hs subfamily located on band 4p15.32 at coordinates chr4:15452168-15458393 (Figure 2a). We isolated 263 sequence tags from this element from lymphoblastoid cells of GM10861 (Additional data file 2), corresponding to 24% of all mapped tags from that individual. An additional 86 tags to this element were isolated from four more individuals (parents GM11994 and GM11995, and the unrelated individuals GM17032 and GM17033; Additional data file 3), indicating that the element at 4p15.32 is highly expressed in lymphoblastoid cell lines. A previous study found that the 4p15.32 element is nearly fixed in four human populations (heterozygosity ≤ 0.05) [49].

The majority of expression tags to this locus end 42 nucleotides downstream of the element (Figure 2b, chr4 short tag), just upstream of a polyadenylation stretch in the genomic DNA. However, two expression tags extend to 182 nucleotides downstream (Figure 2b, chr4 long tag), suggesting that at least some of the transcripts might continue further into the flanking DNA. Directed 3' RACE using a primer located just downstream of the L1 amplified a single product terminating at this same position in both individuals GM11994 and GM11995 [dbEST:64858885]. These 182 nucleotides are also found downstream of another 5' truncated L1 located at chr6: 66316760-66318742 (Figure 2b, chr6 transduction), which was previously described as a member of a transduction family [47]. The chromosome 6 insertion, which is polymorphic in different ethnic populations [49-51], is therefore likely the descendent of the full-length element on chromosome 4. These lines of evidence all point to at least some fraction of the L1 transcript at 4p15.32 terminating 182 nucleotides downstream of the element (Figure 2b, chr4 3' long tag).

The L1 at 4p15.32 contains an intact *ORF1 *gene; however, *ORF2 *is truncated 96 amino acids early, downstream of the known functional domains. The presence of the transduced polymorphic descendent element on chromosome 6 suggests that the 4p15.32 element has been active in the recent past. Nine expression tags from two different individuals were also isolated from a similar sequence (U35) to 4p15.32 that does not occur in the human reference genome (Figure 2b, U35 tag; Additional data files 1 and 3). U35 may represent an allele or an additional non-reference L1 insertion related to the element at 4p15.32.

#### Inter-individual transcriptional polymorphism at 4p15.32

The L1 at 4p15.32 is located in intron 7 of the *CD38 *gene, in the same orientation as the gene (Figure 2a). CD38 (cluster of differentiation 38) is a cell-surface glycoprotein involved in lymphocyte cell adhesion and signaling [52]. We examined steady-state RNA levels of the L1 at 4p15.32 in CEPH familial lymphoblastoid cell lines using a TaqMan quantitative RT-PCR assay specific for the L1 transcript (Figure 2c). Note that expression tags were cloned at high frequency from both GM10861 and GM11994 (Additional data files 1, 2 and 3). There are significant differences in expression among the different individuals, with GM11992 showing little to no expression (Figure 2d), and individual GM10861 showing relatively high expression. We compared the expression of the L1 element to that of the surrounding *CD38 *gene. We found that, while the abundance of the *CD38 *transcript is several orders of magnitude higher, the pattern of expression of the L1 element follows that of expression of the gene (Figure 2e).

#### Characterization of the L1 transcript at 13q14.2

**Figure 3:**
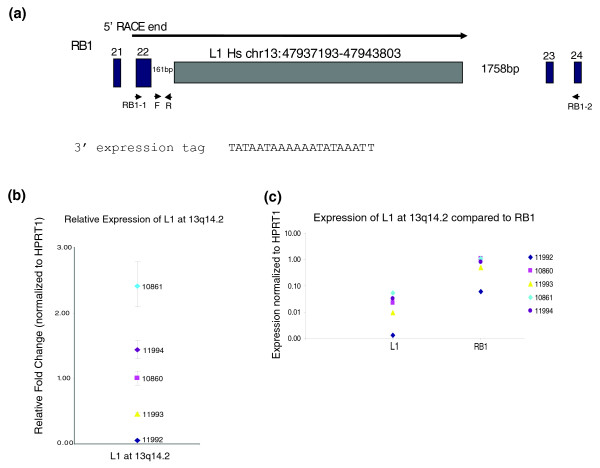
Characterization of L1 at 13q14.2. **(a) **Diagram of L1 at 13q14.2 and the surrounding region. The arrow designates the L1 transcript. Triangles at F and R indicate positions of oligonucleotides 13q14.2F and 13q14.2R. Blue boxes indicate exons of the *RB1 *gene, with exon number designated. Oligonucleotides RB1-1 and RB1-2 are indicated. The sequence of the 3' expression tag is provided. **(b) **Relative expression of the L1 at 13q14.2 in lymphoblastoid cell lines from CEPH individuals. Expression is in arbitrary units normalized to HPRT1. Error bars indicate ± standard deviation from three replicates. **(c) **Expression of CEPH individuals of the L1 at 13q14.2 compared to flanking exons from RB1, normalized to HPRT1. Expression is plotted on a logarithmic scale so that levels for both amplicons can be clearly visualized. Error bars represent ± standard deviations from three replicates. All data are representative of at least two biological replicates.

We also examined three full-length elements that were represented less frequently by 3' expression tags. The L1 element on chromosome band 13q14.2, located at coordinates chr13:47937193-47943803, was represented by six expression tags total cloned from each member of the GM11994/GM11995/GM10861 familial trio (Table 2; Additional data files 2 and 3). The 3' end tags terminate 20 nucleotides downstream of the end of the element, within a poly(A) rich region (Figure 3a). The associated L1, while classified in the human-specific L1Hs subfamily, does not contain intact ORFs and is not expected to be competent for retrotransposition.

The 13q14.2 L1 is located inside intron 22 of the *Retinoblastoma 1 *(*RB1*) tumor suppressor gene [53], in the same orientation (Figure 3a). 5' RACE analysis specific to this locus amplified a single product from individual GM11994 [dbEST:64858883], designating a transcriptional start site 457 bp upstream of the start of the element that encompasses part of intron 21 and all of exon 22 of *RB1 *(Figure 3a). This position is over 161 kb downstream of the start of *RB1*. Real-time RT-PCR analysis in familial cell lines indicates differential expression of the L1 amongst related individuals (Figure 3b), with individuals GM10861 and GM11994, from which expression tags were cloned, showing relatively high expression, and individual GM11992 showing an almost complete absence of expression. Expression of the *RB1 *exons flanking the L1 correlated with expression of the L1 transcript (Figure 3c), although the *RB1 *gene was expressed overall at a higher level (approximately 20 to 50 times more abundant).

L1s are typically cytosine methylated, a modification that is associated with suppression of expression (for example, [29]). We hypothesized that methylation might be lost in expressed elements; therefore, we examined cytosine methylation, both at the start of transcription of the L1 and within the L1 5' UTR, in two cell lines (GM10860 and GM11992; Figure 3b) that showed stark differences in expression levels. In both cases, the sequences were densely methylated (see Materials and methods), indicating a lack of an obvious role for cytosine methylation in quantitative expression differences at this site.

#### Characterization of the L1 transcript at 6p22.2

**Figure 4:**
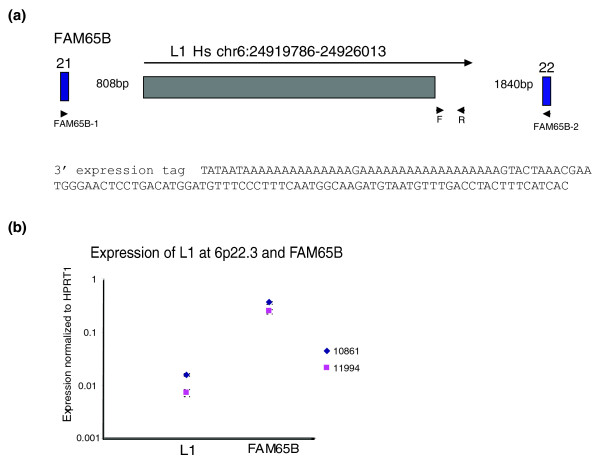
Characterization of L1 at 6p22.2. **(a) **Diagram of L1 at 6p22.2 and the surrounding region. The arrow designates the L1 transcript. Triangles at F and R indicate positions of oligonucleotides 6p22F and 6p22R. Blue boxes indicate exons of the *FAM65B *gene, with exon number designated. Oligonucleotides FAM65B-1 and FAM65B-2 are indicated. The sequence of the 3' expression tag is provided. **(b) **Expression of CEPH individuals GM10861 and GM11994 of the L1 at 6P22.2 compared to flanking exons from *FAM65B*, normalized to HPRT1. Expression is plotted on a logarithmic scale so that levels for both amplicons can be clearly visualized. Error bars represent ± standard deviations from three replicates. Data are representative of two experimental replicates.

The L1Hs element at 6p22.2, genome coordinates chr6:24919786-24926013, was represented by a single 3' expression tag from individual GM17032 (Table 2; Additional data file 3). However, this element had been previously found to be one of the most retrotranspositionally active in a cell culture assay, as well as polymorphic in natural populations [19,54]. As expected from an element that is known to be active, both ORFs are intact. The 3' expression tag terminates 119 nucleotides downstream of the element (Figure 4a). 3' RACE analysis in two unrelated individuals (GM11994 and GM11995) using a primer immediately next to the L1 identified a single product terminating 442 nucleotides downstream of the L1 [dbEST:64858886]. Therefore, the transcript from this L1 can transduce at least 442 bp of genomic sequence.

The L1 at 6p22.2 is located within intron 21 of the *FAM65B *gene (Figure 4a), which encodes a factor involved in trophoblast differentiation [55]. Expression of the element was confirmed in lymphoblastoid cell lines from individuals GM10861 and GM11994, both of which are heterozygous for this insertion. The element in GM10861 was expressed greater than two-fold more than in GM11994 (Figure 4b). Transcripts corresponding to the *FAM65B *gene, while more abundant than those from the L1 (approximately 30 times), appear to correlate with expression of the L1 in these two individuals (Figure 4b).

#### Characterization of the L1 transcript at 1p22.3

**Figure 5:**
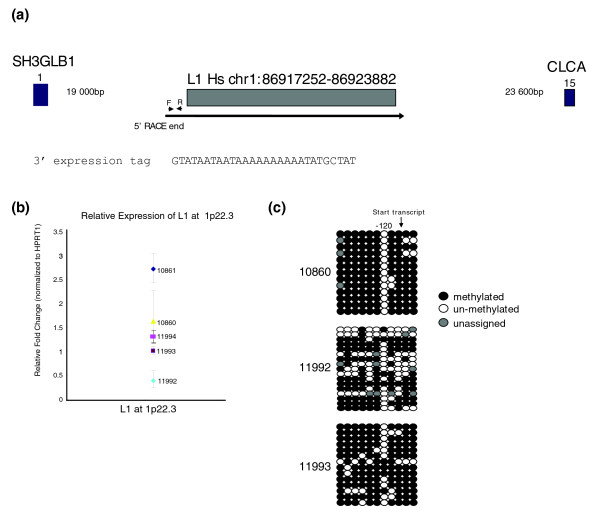
Characterization of L1 at 1p22.3. **(a) **Diagram of L1 at 1p22.3 and the surrounding region. The arrow designates the L1 transcript. Triangles at F and R indicate positions of oligonucleotides 1p22.3F and 1p22.3R. Blue boxes indicate exons of nearest genes *SH3GLB1 *and *CLCA*, with exon number designated. The sequence of the 3' expression tag is provided. **(b) **Relative expression of the L1 at 1p22.3 in lymphoblastoid cell lines from CEPH individuals. Expression is in arbitrary units normalized to HPRT1. Error bars indicate ± standard deviation from three replicates. All data are representative of at least two biological replicates. **(c) **Bisulfite sequencing of amplicon surrounding the start of transcription of the L1 at 1p22.3 in GM10860, GM11992 and GM11993. Each circle represents a single CpG, while each row is an individual sequence clone. The position of the start of transcription as determined by 5' RACE is indicated. Filled circles indicate lack of bisulfite conversion - that is, cytosine methylation. Grey circles indicate residues that could not be assigned due to sequence polymorphism or poor sequence quality. The CpG at position -120 is unmethylated in the majority of clones from all three individuals.

The L1 at 1p22.3, genomic coordinates chr1:86917252-86923882, was identified by a single 3' expression tag cloned from individual GM10861 (Table 2; Additional data file 2). This element, a member of the L1Hs subfamily, encodes two intact ORFs, and therefore might be retrotranspositionally competent. In contrast to the three elements characterized above, the L1 at 1p22.3 is intergenic, located 19 kb and 23.6 kb from its nearest neighboring genes (Figure 5a).

The 3' expression tag corresponding to this locus ends 27 nucleotides downstream of the end of the L1 (Figure 5a). Directed 5' RACE from cell line GM11994 identified a single transcriptional start site 125 bp upstream of the element [dbEST:64858884] (Figure 5a). The L1 transcript shows some variant expression within lymphoblastoid lines from two CEPH families (GM10860/GM11992/GM11993 and GM10861/GM11994; Figure 5b). Bisulfite sequencing analysis on this trio indicates differential methylation levels, with GM11992 showing the least CpG methylation (Figure 5c). Because this is also the individual expressing the least transcript, cytosine methylation does not appear to correlate with transcriptional repression at this site.

## Discussion

We have found evidence for transcription of 692 distinct L1 element sites in human lymphoblastoid cells. Of these, 410 sites correspond to full-length elements (Table 1), including 52 of the 304 human-specific subfamily (Tables 2 and 3). Of the sixteen full-length human-specific elements that we identified carrying intact copies of both *ORF1 *and *ORF2*, only five were represented by five or more expression tags (Tables 2 and 3). Therefore, while many L1 elements are transcribed in somatic cells, paradoxically few are likely to be active.

The proportion of full-length elements that we identified containing intact ORFs was not significantly different from their occurrence in the genome, suggesting that transcription of putatively functional elements is tolerated in somatic cells. Only six of these intact L1s corresponded to those that are known to be highly active in a cell culture assay (Tables 2 and 3) [19], suggesting that the most retrotranspositionally competent elements may be suppressed in somatic cells. Alternatively, the individuals that we assayed might carry less active alleles of these previously examined elements (see [56,57] for evidences of known allelic variation in L1s). Six expressed full-length elements encode intact *ORF2 *in the absence of *ORF1 *(Tables 2 and 3). These elements would not be able to mobilize themselves in *cis*, but could possibly retain the ability to mobilize non-autonomous elements such as Alus in *trans *[21].

A previous study examining transcription of the native promoter of new L1 insertions in cell culture found that 5' transcript ends typically mapped within a few base pairs of the 5' end of the element [12]. Our data suggest that the transcription start site of endogenous, evolutionarily older elements may also begin further away from the element. Half of the sites identified by 5' RACE mapped within 50 nucleotides of the start of the L1 element as indicated by sequence homology. We also found over 100 L1 transcription start sites situated greater than 100 nucleotides upstream of the element. These start sites might not result from action of the L1 promoter at all, but instead use another promoter located coincidentally in the flanking genomic vicinity of the element. This hijacking by the L1 of an upstream promoter might be advantageous for an element whose native promoter has either degenerated through mutation or been subjected to epigenetic silencing.

Examination of the expression of four full-length human-specific elements in members of a CEPH Utah family indicated a high degree of variation among different individuals. This is consistent with previous studies showing inter-individual and inter-allelic variation in retrotransposition of different highly active L1 elements [56,57]. A larger population study is required to determine how widespread transcript-level variation is among different individuals and whether this variation is genetically tractable. Some individuals, such as GM10861, showed increased expression, while other individuals, such as GM11992, showed little to no expression at all four loci. These differences were not noted at the *HPRT *or 18S loci (data not shown; see Materials and methods), which were evenly expressed in all individuals. We hypothesize that there might be a genome-wide level of regulation leading to individuals with higher or lower numbers of expressed L1 elements. We expect that further, more expanded studies in different human populations will reveal a great amount of natural variation in the number and location of L1s contributing to the human transcriptome.

Transcription of L1s within genes might be expected to interfere with transcription of the gene, so that genes containing highly expressed retroelements in their introns might be relatively suppressed. By contrast, we found that expression of the L1 elements at 4p15.32, 13q14.2, and 6p22.2 closely mirrored the expression of surrounding spliced exons from protein-coding genes. In the case of 13q14.2, a transcription start site was identified 457 nucleotides upstream of the L1, tens of kilobases downstream of the start of the *RB1 *gene. Therefore, it is unlikely that action of the *RB1 *promoter itself contributes directly to regulation of the L1. Instead, we hypothesize that transcription of *RB1 *results in the creation of a region of open chromatin that facilitates the activity of other promoters located within that region. Alternately, both loci might be located in a larger, transcriptionally permissive epigenetic domain. We note that Faulkner *et al*. [41] also found a positive correlation in expression between transcripts originating in retroelements and surrounding genes.

Regardless of whether the L1 transcripts are produced through the action of their native promoters or an upstream promoter, the transcripts identified through 3' end tagging terminate near the end of the L1. The short transductions that we identified most likely use the L1-encoded polyadenylation signal. In a few cases, longer transductions that were greater than 50 nucleotides were seen; this is consistent with previous studies describing L1 elements carrying transductions of their progenitor locus [44,46,47]. Where elements are located within genes, such as the elements at 4p15.32 and 6p22.2, a transcript originating from the gene may terminate prematurely by polyadenylation at the end of the L1. In this way, the intronic L1 transcript might 'break' the expression of downstream exons [23]. However, as the L1-incorporating transcripts described in this study are expressed at much lower steady-state levels than the surrounding genes, it is unclear as to what extent their termination influences the expression or function of those genes.

Cytosine methylation is known to suppress the activity of endogenous L1 promoters [27,29]; our examination of the regions surrounding the start of transcription of two elements (the L1s at 1p22.3 and 13q14.2) did not find strong evidence for this modification modulating individual-specific expression levels. Other factors, such as histone modifications, nucleotide polymorphisms, and *trans*-acting transcriptional regulators may function at these loci as rheostats to specify the exact levels of RNA production in different cells, tissues, and individuals. Moreover, differences in post-transcriptional regulation, for instance, through sequestration into subcellular compartments [58,59], may further determine which full-length, putatively functional L1s are able to actively retrotranspose.

Our study is limited in that we are only able to detect transcripts that carry unique non-L1 sequence at either the 3' or 5' end. In addition, we have only sampled a single individual at the 5' end and a single individual in depth at the 3' end, in a single tissue type - transformed lymphoblastoid cell lines. Because of these caveats, we expect that we have found a subset of the transcribed element sites in different human tissues and populations. Indeed, we note that only 42 of the 606 L1 sites that we identified were also identified by an independent high throughput study looking for transcription start sites mapping within transposable elements [41] (Additional data files 1 and 4), a study very different from ours in terms of methodology, individuals and cell types.

In addition to the major L1 sense promoter, L1s also contain an antisense promoter in the 5' UTR that produces transcripts of the upstream flanking region [60-62]. A recent study has also found evidence for an additional outward-facing sense promoter in the 3' UTR [41]. As such, explorations of sense transcription through the L1 may reveal only the tip of the iceberg of the genomic transcripts incorporating and influenced by the presence of these retroelements.

## Conclusions

We have identified expressed sequence tags corresponding to 692 distinct L1 element sites in human lymphoblastoid cells, indicating that retrotransposon-derived sequences contribute to the transcriptional output of somatic cells. 410 sites correspond to full-length elements in the human reference genome, of which 52 are from the most evolutionarily recent L1Hs subfamily. Closer examination of four L1Hs subfamily full-length elements revealed significant variation in expression levels within a single family. Therefore, many L1s are expressed, and this expression can differ in different individuals. We look forward to more in-depth investigations of L1 expression polymorphisms in human populations and the potential influence of this variation on the function of adjacent genomic regions.

## Materials and methods

### Cell culture and nucleic acid extraction

Cell lines from the CEPH Utah pedigrees (GM10860, GM10861, GM11992, GM11993, GM11994, GM11995) and GM17032, GM17033 and GM17045 are all part of the National Institute of General Medical Sciences human genetic cell repository (Coriell Institute for Medical Research). Cells were grown in RPMI (Life Technologies, Carlsbad, CA, USA) supplemented with 15% fetal bovine serum (Thermo Fisher Scientific, Waltham, MA, USA), 1% L-glutamine (Life Technologies) and 1% penicillin/streptomycin (Life Technologies) at 37°C/5% CO_2_. Cultures were maintained at concentrations of 0.25 to 1 × 10^6 ^cells/ml.

Genomic DNA was isolated using the DNeasy Tissue Kit (QIAGEN, Valencia, CA, USA). Total RNA was isolated with the RNeasy Mini Kit (QIAGEN). For expression tag isolation, polyA+ RNA was isolated from total RNA using the Oligotex Direct mRNA Mini Kit (QIAGEN). RNA aliquots destined for cDNA synthesis were treated with DNaseI (Life Technologies, Carlsbad, CA, USA).

### Expression tag isolation

For 3' expression tag isolation, first strand cDNA was produced from polyA+ RNA using MMLV reverse transcriptase (Ambion, Austin, TX, USA), primed with either 3' RACE adaptor or FusionBRACE adaptor. This product was subjected to two rounds of PCR (iTaq, Bio-Rad, Hercules, CA, USA) using oligonucleotides L1HsSP1A/3' RACE outer for 40 cycles for the first round and L1HsSP3A/3' RACE inner or FusionAL1Hs/FusionBRACE inner for 40 cycles for the second round. First strand product was purified through a QIAGEN PCR Purification column prior to the second round. Second round product was run on an agarose gel and purified using the QIAGEN Gel Extraction Kit. Alternatively, linear PCR (Fast Start High Fidelity, Roche, Basel, Switzerland) was conducted for 35 cycles on the first strand cDNA using FusionAL1Hs without a reverse primer. Resulting product was purified through a QIAGEN PCR Purification column.

5' RACE first-strand cDNA was primed using 5' RACE adaptor using the First Choice RLM RACE Kit (Ambion). The 35-cycle first round of PCR (iTaq, Bio-Rad) was conducted using 5' RACE outer and L15PUR, and the product was purified using a QIAGEN PCR Purification column. The 40-cycle second round of PCR (Fast Start High Fidelity, Roche) was conducted on first round product using FusionAL15RACE and FusionBRACEinner. This product was gel isolated using the QIAGEN Gel Extraction Kit, purified a second time through a PCR Purification column, and sent to the DNA Sequencing Facility at the University of Pennsylvania School of Medicine for 454 pyrosequencing (Roche) (see below).

5' and 3' RACE specific to the L1s at 4p15.32, 13q14.2, 6p22.2, and 1p22.3 were conducted using the First Choice RLM-RACE Kit (Ambion) according to standard protocols. Two rounds of PCR were conducted using either iTaq (Bio-Rad) or Fast Start High Fidelity enzyme (Roche). First round 5' RACE used oligonucleotides L15PUR/5' RACE outer, while first round 3' RACE used oligonucleotides L1HsSP1A/3' RACE outer. These primer sets amplify L1 sequence indiscriminately. Second round PCRs used corresponding 5' or 3' RACE inner primers along with element site-specific 3' RACE or 5' RACE primers. Sequences from locus-specific 5' and 3' RACE were deposited in dbEST [63]; accession numbers are given in the Results section.

Refer to Additional data file 5 for all oligonucleotide sequences.

### Bisulfite analysis

Genomic DNA was treated with sodium bisulfite using the Imprint DNA Modification Kit (Sigma-Aldrich, St. Louis, MO, USA). Amplicons from L1s located near 13q14.2 and 1p22.3 were generated using iTaq. Oligonucleotide sequences can be found in Additional data file 5. At least 13 clones were sequenced from each amplicon in each individual. More than 99% of cytosines located in non-CpG contexts were converted, indicating effective chemical modification.

For the 13q14.2 amplicon near the L1 transcription start site, individuals GM10860, GM11992 and GM11993 featured 100%, 100%, and 98.5% CpG methylation, respectively. For the 13q14.2 amplicon located in the L1 5' UTR, GM10860 and GM11992 featured 96.7% and 97.9% CpG methylation, respectively. GM10860, GM11992, and GM11993 featured 86.4%, 57.2%, and 81.8% CpG methylation in the vicinity of the 1p22.3 L1 transcription start site. Figure 5c shows a visual representation of the data for the 1p22.3 amplicon.

### Cloning and sequencing

Purified PCR amplicons from expression tagging, RACE and bisulfite analysis were cloned into PCR4. TOPO (Invitrogen) and transformed into TOP10 cells (Invitrogen), and plated on LB (lysogeny broth) supplemented with 50 μg/ml kanamycin and 20 μg/ml X-gal at 37°C overnight. Single colonies were amplified in LB plus 50 μg/ml kanamycin liquid culture, and plasmid was isolated using the QIAGEN Plasmid Mini Kit. Alternately, colonies were propagated in 220 μl of LB/10%glycerol/50 μg/ml kanamycin in 96-well culture dishes for high-throughput plasmid isolation and sequencing. Sanger sequencing was conducted by the DNA Sequencing Facility using the M13-forward primer located on the vector backbone.

### 454 pyrosequencing

5' RACE PCR products were initially cloned and subjected to Sanger sequencing (see above) prior to 454 pyrosequencing. We identified 147 5' RACE amplicons representing 58 L1 sites in the reference genome by this pilot Sanger sequencing project. Almost all of these sites were later corroborated in the 454 pyrosequencing; as a result, we only report the 454 results in Table 1 and Additional data file 4.

Pyrosequencing was performed on a Roche/454 GS FLX machine by the University of Pennsylvania DNA Sequencing Facility, according to procedures recommended by the manufacturer. Briefly, emulsion PCR was conducted to amplify DNA from a single bead-bound copy to millions of copies per bead in an emulsion of water-in-oil mixture. Beads carrying amplified DNA were isolated from empty beads based on the binding of biotinylated amplification primers to streptavidin. Sequencing primer B was then annealed to the bead-bound amplicons, resulting in sequence generation that proceeded from the 5' RACE-identified transcription start site towards the L1. Beads were loaded into the wells of a picotiter plate such that the wells contain single DNA beads. The picotiter plate was then loaded with packing and enzyme beads and inserted into the FLX instrument and the sequencing reagents were sequentially flowed over the entire plate. Pyrophosphate released after incorporation of a nucleotide into the growing DNA strand by DNA polymerase was detected by ATP sulfurylase and luciferase, with the light signal recorded by a CCD camera from every well on the plate simultaneously in a massively parallel fashion. More detailed methods can be found at [64].

5' RACE PCR for individual GM11994 was run on one-quarter of a picotiter plate, resulting in 38,602 reads; 2,514 reads did not match sequencing primer B sequence and were discarded. The reverse primer FusionBRACEinner (or any substring of the reverse primer down to 5 bp) was trimmed from the ends of the 36,088 remaining reads. Refer to Additional data file 6 for a compilation of mapped 454 sequencing reads.

### Quantitative RT-PCR

Total RNA was treated with RQ1 RNase-Free DNase (Promega, Madison, WI, USA) and cDNA synthesized using the Applied Biosystems (Foster City, CA, USA) High-Capacity cDNA Reverse Transcription Kit. Typically, 2 μg of total RNA was added to a 20 μl first strand synthesis reaction.

The SYBR green method was used for most amplicons (SYBR Green PCR master mix (2×); Applied Biosystems), in conjunction with oligonucleotide pairs F/R as indicated (Figures 2, 3, 4 and 5; Additional data file 5). The exception was the L1 at 4p15.32, which was assayed with the TaqMan method (TaqMan Gene Expression master mix, Applied Biosystems) using the oligonucleotide pair 4p15.32R/L1HsSP1A and the antisense strand 4p15.32 TaqMan probe (Additional data file 5). Quantitative PCR was carried out in 20 μl of total volume reactions containing 150 nmol of primers and 1 μl of RT reaction. Amplification reactions were performed in the Applied Biosystems 7900 HT Fast Real Time PCR System following the manufacturer's instructions with the following conditions: denaturation program (95°C for 10 minutes) and amplification program repeated 40 times (95°C for 20 s, 51°C to 57°C for 30 s, 72°C for 30 s). The PCR protocol was optimized for the different Tm of the oligonucleotides. The data were exported from the 7900 HT Sequence Detection System Software v2.2 into a Microsoft Excel spreadsheet. Expression levels in arbitrary units were calculated relative to amplification of HPRT1 using the ΔCt method followed by a log_2 _transformation. Mean values and standard deviations were calculated based on the three replications per sample. At least two biological replicates were conducted for most amplicons; data in Figures 2, 3, 4 and 5 are representative. Experiments were also conducted using 18S as an internal reference control, with the results of the analysis not significantly different from those with HPRT (data not shown). Oligonucleotide primers for quantitative PCR were designed using Primer3 [65].

All individuals subjected to RT-PCR analysis were initially genotyped for presence of the L1 insertion. The individuals were found to be homozygous for the insertion unless otherwise noted in the Results section (data not shown). Preliminary semi-quantitative RT-PCR was also conducted using iTaq (Bio-Rad) and oligonucleotides located within the expression tags (data not shown) to confirm that all amplicons could be amplified sufficiently in less than 40 cycles of PCR to be visualized by standard ethidium bromide staining. Please refer to Additional data file 5 for the sequences of oligonucleotides located in the vicinity of L1s at 4p15.32, 13q14.2, 6p22.2 and 1p22.3.

### Bioinformatics analysis

The genomic origin of 3' expression tag sequences was mapped using Tagscan [66] or BLAT [67]. Tags were only assigned to a particular location if the alignment contained fewer than three mismatches, not counting within the primer sequence. Sequence tags with multiple equally good matches were not assigned to a location. Sequences with no good matches were considered to be not present in the reference genome and were designated U1 to U43 (Additional data files 1, 2 and 3).

5' RACE trimmed reads from 454 pyrosequencing were aligned to the human genome reference using BLAT [67] (Additional data file 6). The best single hit was determined by examining the match with the greatest value for number of matched minus number of mismatched bases, and assigning that sequence to the nearest L1 element. While most of this analysis used a script developed in-house (Adam D Ewing), BLAT hits that were not near a primate-specific L1 were curated manually.

Human genome information, including L1 annotation information (for example, numbers in each subfamily, sequences, orientations relative to genes), was obtained from the UCSC Genome Browser [68,69], with all coordinates corresponding to hg18 (March 2006). Previously known polymorphic L1 insertion sites are found in the dbRIP database [16].

## Abbreviations

CEPH: Centre d'Etude du Polymorphisme Humain; L1: LINE1 (long interspersed nuclear element); ORF: open reading frame; RACE: rapid amplification of cDNA ends; UTR: untranslated region.

## Authors' contributions

SHR and HHK conceived the study. SHR collected, compiled and analyzed the data and drafted the manuscript. LZ conducted quantitative PCR analysis. All authors examined and approved the final manuscript.

## Additional data files

The following additional data are available with the online version of this paper: a spreadsheet listing the total 3' expression tagged sites from individuals GM10861, GM11994, GM11995, GM17032, GM17033, GM17045 (Additional data file 1); a spreadsheet listing the subset of 3' expression tagged sites from individual GM10861 only (Additional data file 2); a spreadsheet that lists 3' expression tagged sites from individuals GM11994, GM11995, GM17032, GM17033, and GM17045 separately (Additional data file 3); a spreadsheet presenting 5' RACE tagged sites from individual GM11994 (Additional data file 4); a spreadsheet listing oligonucleotides used in this study, as well as sequence corresponding to the L1 breakpoint region of a 5' RACE expression tagged site to a non-reference L1 (Additional data file 5); a csv file of 454 5' expression tag pyrosequencing sequence reads and their putative genomic matches (Additional data file 6).

## Supplementary Material

Additional data file 1 The spreadsheet includes cDNA sequence, L1 assignments, tag counts and related information as indicated in the header. Click here for file

Additional data file 2 The spreadsheet includes cDNA sequence, L1 assignments, tag counts and related information as indicated in the header. Click here for file

Additional data file 3 The spreadsheet includes sequences, L1 assignments, counts, and related information. Click here for file

Additional data file 4 The spreadsheet includes locations, counts, L1 assignments, and other related information. A second worksheet presents this information for tags corresponding to full-length elements only. Grey font indicates 3' truncated elements, green font indicates splicing from greater than 15 kb upstream, while blue font indicates putative non-reference L1s. Click here for file

Additional data file 5 Oligonucleotides used in this study, as well as sequence corresponding to L1 breakpoint region of a 5' RACE expression tagged site to a non-reference L1. Click here for file

Additional data file 6 454 5' expression tag pyrosequencing sequence reads and their putative genomic matches. Click here for file
